# The Report of the London Committee of Associated Apothecaries and Surgeon-Apothecaries of England and Wales

**Published:** 1813-10

**Authors:** 


*For the Medical and Physical Journal.*

THE REPORT *of the* LONDON COMMITTEE *of* ASSOCIATED APOTHECARIES *and* SURGEON-APOTHECARIES *of* ENGLAND *and* WALES; *with the Resolutions proposed as the Bases of a new Bill intended to be introduced in the next Session of Parliament; and the Correspondence of the London Committee with the Executives of the Royal Colleges of Physicians and Surgeons, and of the Society of Apothecaries*.*

THE London Committee of Associated Apothecaries and Surgeon-apothecaries of England and Wales have, from the period at which the former Bill for "Regulating the Practice of the Apothecary and Surgeon-apothecary" was withdrawn, been sedulously occupied in the investigation of the objections that were urged against that Bill in particular, and against the measure in general; with a view faithfully to fulfil the high duties of the trust reposed in them—to promote the public good—and, by securing a more complete medical education, eventually to render the profession and its practitioners worthy of each other. That their constituents may have full means of judging how far the Committee have accomplished the great objects upon which they have so anxiously deliberated, it has been deemed proper to submit to them the RESOLUTIONS which have been the result of those deliberations, accompanied with the correspondence that has taken place with the medical corporate bodies.

During the last year, the Committee had frequent and extensive communications with every rank in society, to whom the late Bill was of any interest; particularly with many members of parliament: and it was a concurring opinion that, if the second reading of that Bill had been persisted

---

* It is respectfully requested, that the contents of this Report, and the Copies of the Resolutions, be distributed in the country districts. But the Committee beg leave generally to refer to the Monthly Medical Journals, through which, as a medium in very general circulation, they will regularly communicate all their future proceedings to the medical public.

sisted in and carried, it would ultimately have been lost, or so altered in the Committee of the House of Commons, as to have fallen very short of the purposes for which it was solicited.

Independently of the opposition of three bodies, two of which had confessedly powerful interest, it was represented to be constructed more with a view to private advantage than public benefit; that it was encumbered with many different (perhaps discordant) objects; and that, being at once narrow in principle and complex in form, it could not be countenanced by parliament.

The observations of the Committee, confirmed by the ablest writers who have traced the causes of the present state of the avocation of the apothecary and surgeon-apothecary, clearly and forcibly show that the true source of the evils of which they justly complain, and for the redress of which they associate, is to be found in the facility afforded, by existing circumstances, to every pretender and unlearned person for assuming the name, character, and functions of that department of the medical profession; and from the total want of all means, either prescriptive or legislative, for punishing, or even exposing, such dangerous intruders.

With these facts before them, aware of the jarring complexity of the former Bill, to disarm opposition by avoiding extraneous interests, to acquire general approval by candid avowals, and to guard against misapprehension by simplicity, the Committee have founded the bases of the new Bill on few and nearly self-evident propositions, unequivocal of honest zeal for the *public health.*

Impelled by an anxious desire to remove every impediment to the arrangement of a Bill, comprehensive, yet simple, but efficient for its purposes—the Committee have witnessed, with deep regret, that many of their supporters and correspondents have been too solicitous in pointing out local grievances, and the means of removing them, more calculated to individual and immediate interests, than regardful of the radical defects in the system of education; and which is so justly and truly alleged to be a principal cause of the present deteriorated state of the practice of the apothecary and surgeon-apothecary.

Sensible, however, of the necessity to abandon all minor considerations, and leave local grievances to be corrected, and immediate and individual interests guarded by a few simple but comprehensive principles—the Committee have formed a series of resolutions, now submitted to the consideration of the medical public, as the bases of a new bill, which they hope will be found to embrace all the essential points; points; and which, when sanctioned by the legislature, will gradually, but certainly, reform that branch of the profession comprehended under the terms apothecary and surgeon-apothecary, rendering it more respectable, and consequently more advantageous, both to the community at large and to the practitioner.

When the comments of the District Committees upon these Resolutions shall be received, the whole will undergo a careful revision, and will be laid before counsel, to receive the appropriate form and legal detail.

A perusal of the correspondence of the Committee with the medical chartered bodies, will show and record the ill success that has attended the repeated and respectful overtures made by the Committee to the Royal College of Physicians of London; and that the Royal College of Surgeons, and the Society of Apothecaries, have returned no answer to the addresses sent to those bodies some weeks since.*

Under these circumstances, it is, at present, impossible to suggest the form, the source, or the constitution of a superintending body; but on this, as on many other important parts of the arrangement, the Committee recommend a confident reliance on the wisdom of parliament.

Events have justified the diligence of the Committee in endeavoring to provide a fund which should be fully adequate to meet all contingencies, and insure the security of the object they have in contemplation, against the chance of failure for the want of means. The gross of the sums received, and the amount of those reported from the country as actually subscribed, induced a well-grounded conclusion, that the resources were equal to every expenditure that could be reasonably anticipated. Consonant with that frankness and desire of communication which the Committee always professed, they expended about 500*l.* in printing, advertisements, postage, &c. &c. that every practitioner in England and Wales might become acquainted with, and be invited to unite in, one great and general Association. In every particular the Committee have endeavored to observe the strictest economy in their expenditure; but, notwithstanding these precautions, and that the Bill passed through one only, and that the least expensive stage, the aggregate expenditure to the present date amounts to 770*l.* 8*s.*

In the general statement subjoined, it is shown that the balance in the treasurer's hands is only 1221*l.* 8*s.* 10*d.*

* The Address to the Royal College of Surgeons was dated July 7th; that to the Society of Apothecaries, August 5th, ultimo.

When

When it is considered that all the parliamentary and legal charges attending a new Bill, must begin *de novo*, it is certain, if any opposition be encountered, that the present fund will be too small to sustain the expenses of so vast an undertaking with any reasonable hope of success. It is, therefore, an object of the highest moment, that it be promptly and liberally replenished.

The practitioners of London and its environs, who do not constitute a twelfth part of those of England and Wales, have raised above a third of the subscription; and many of the most eminent have devoted a very considerable portion of time, of labor, and of private cost, in assisting the cause.

If the sums which were subscribed in the country, the number and amount of which were transmitted to the London Committee, with assurances of being speedily remitted, had been duly received, the necessity for this further appeal for pecuniary aid would not have been experienced. In this particular, great disappointment and some surprise has been felt. In some instances, the subscriptions have been, without any reason being assigned, entirely withheld; in others, partially remitted; some have made deductions for personal and public expences incurred by them, of twenty-five, thirty, and even fifty per cent. from the aggregate amount of the subscription of entire districts; some keep back money for expences that *may* arise; and many individuals have altogether omitted to fulfil their promises!

However reluctantly, the Committee, in the due execution of their trust, are compelled to acknowledge these disagreeable truths; nor could they discharge the duty they owe to themselves and their constituents, did they not make a full representation of a difficulty, which, if not overcome by more unanimous liberality, and more just and generous feelings, must render nugatory a plan formed to restore and to render permanent the respectability of this branch of the medical profession, and to afford an extensive benefit to society.

If the efforts of the Committee deserve support, they must be met with prompt and vigorous exertions on the part of the country practitioners; subscriptions must be renewed, and those who have not yet subscribed must be solicited. But it is not by subscribing to the fund only that the country practitioner is now called upon to further this great cause. The recess of parliament will afford to him ample opportunity to ascertain the sentiments and receive the advice of members of the House of Commons; to impress on them a full and clear understanding of the motives, the objects, the operation, and the effects of this application to the legislature. Every chance should be seized of explaining to the peers of the realm, as well as the members of the House of Commons, the benefits that must result to all descriptions of persons by this measure; the little possible evil that can arise out of its operation; and the preponderance of good that will necessarily be the result of its passing into a law.

Neither can the Committee omit to remark, that the present is the time for removing the prejudices which have been so industriously excited in the public mind; nor can it be too often enforced, that, if there were errors in the former Bill, they were those of judgment, and unavoidable in a measure for which no precedent afforded a guide. The Resolutions, as outlines of the intended Bill, should be submitted to members; their opinion should be consulted, and their interest and support engaged. It is of the utmost importance likewise, that the names of such members who declare their good-will toward the measure, should be punctually sent to the chairman of the London Committee.

The Committee, anterior to the meeting of the country deputies on the 23d of March, had received many sensible and highly-interesting communications on that important subject—*Medical Attendance on Parochial Poor;* a system which every moral and religious obligation demands should be amended. Since that period, nothing of importance on the subject has been added.

It appears indeed a serious evil to the poor, and a just cause of complaint among country practitioners. But the London Committee avow their incompetency to suggest a remedy, though they will exert their utmost interest to promote any generally approved plan that may be proposed. That both the evil and the remedy may receive all the elucidations which they so strongly claim, it is strenuously advised that every opportunity be sought of directing the attention of members of parliament, to inquire, personally, and investigate the real sources of the nefarious and disgraceful scenes which are perpetually, and almost universally, passing in every country parish, in which the health and comfort of the poor are the certain sacrifice. This will be laying the surest foundation for redress.

Finally, the Committee recommend, most earnestly, that a petition be written *(not printed)* on parchment, agreeably to the form herewith given, and laid on the table of every county, city, or district medical meeting, throughout England and Wales, for the signatures of those present; that it be conveyed to neighbouring parishes by some confidential person for a similar purport; and that every individual subscriber should not only state his place of residence, but if he be be a member of the Royal College of Surgeons, or of the Society of Apothecaries, to insert that distinction; and that when the petition has received all the signatures expected, it be formally requested of the representatives for the county, city, or borough, to present and support the same by their presence and votes in parliament when leave has been given for the introduction of the Bill.

The Committee have endeavored as briefly and perspicuously as possible to inform the medical public, in this Report, of the steps they have with undeviating steadiness pursued to attain the important object for which they were elected. In the execution of the task, they have had much prejudice to combat, many discordant opinions to reconcile, and have experienced no small degree of obloquy. They have persevered, however, conscious of the integrity of their intentions; nor do they entertain the least doubt of seeing their labors crowned with success. But they are aware, success depends not on their exertions only. Reflection, and a dispassionate retrospect of the past, must have convinced the supporters of the late Bill that any one formed on such principles, and embracing such complicated views, would never meet the approbation and countenance of the legislature, and must always encounter a formidable and dangerous opposition.

The Committee, therefore, submit the following Resolutions for consideration, in the confident hope that they will be found entitled to general approval and support.

*The Resolutions proposed by the London Committee as the Bases of a new Bill.*

1st.—That it shall not, in future, be lawful for any person, except those already in practice, to act as an apothecary, surgeon-apothecary, or as a practitioner in midwifery, in any part of England or Wales, unless such person shall have been first examined, and received a certificate of his being duly qualified for such practice: provided always, that no person shall be entitled to such examination until he has attained the age of twenty-one years.

2d.—That no person, excepting such as are actually indented, or have commenced a course of medical studies, at the time of passing this act, be admitted to an examination for a certificate to practise as an apothecary or surgeon-apothecary, unless he has served an apprenticeship of not less than five years to an apothecary or surgeon-apothecary, and shall produce other testimonials of a sufficient medical education.

3d.—That no person be permitted to practise as an apothecary, thecary, either alone or conjointly as a surgeon-apothecary, unless he has been examined as to his knowledge of medicine and pharmacy, by a board of medical practitioners, properly qualified and legally authorised for that purpose, and likewise for the purpose of examinations in midwifery.

4th.—That no person acting or having acted as full surgeon or apothecary in the army or navy, shall be liable to an examination, except as to his qualification in midwifery.

5th.—That no person, in future, shall be allowed to practise surgery alone, or conjointly with pharmacy and midwifery, until he shall have obtained a diploma from the Royal College of Surgeons.

6th.—That no person, in future, act as an assistant to an apothecary, or surgeon-apothecary, to compound and dispense medicines, without passing an examination in pharmacy, unless he shall have served an apprenticeship of five years to an apothecary or surgeon-apothecary.

7th.—That no female, in future, be allowed to practise midwifery, without passing an examination.

8th.—That every apprentice's indenture shall bear a stamp of twenty-five pounds.

9th.—That nothing herein contained be considered as preventing members of the Royal College of Physicians, or of the Royal College of Surgeons, or of the Society of Apothecaries, of London, enjoying the same privileges, and immunities, in their several branches of the profession, to which they are at present entitled.

(Signed) GEORGE MAN BURROWS,

*Bloomsbury-square,* Chairman.

*Sept.* 4, 1813.

N.B.—All communications and subscriptions are requested to be addressed to the chairman, by the first week in November. The petitions should be prepared and signed by the opening of the next session, and be ready to be presented to parliament when the Bill has been read the first time.

---

*State of the Treasurers' Accounts of the Association of Apothecaries and Surgeon-Apothecaries, from June 30th,* 1812, *to September*, 1813.

| | £. | s. | d. |
|---|---|---|---|
| Subscriptions received | 1956 | 14 | 10 |
| Return from sale of Reports and Abstracts of Bill | 35 | 2 | 0 |
| | £1991 | 16 | 10 |

| Disbursements as undermentioned: | £. | s. | d. |
|---|---|---|---|
| Printing and copying | 316 | 3 | 0 |
| Clerk and collector | 32 | 3 | 6 |
| Advertisements | 101 | 18 | 1 |
| Public and Committee meetings | 44 | 6 | 0 |
| Solicitor's bill for legal and parliamentary charges | 185 | 15 | 0 |
| Sundry petty charges, including paper, postage, carriage of parcels, &c. | 90 | 2 | 5 |
| | 770 | 8 | 0 |
| Balance in treasurers' hands | 1221 | 8 | 10 |
| | £1991 | 16 | 10 |

N.B.—Of this balance, 1011*l.* 14*s.* 5*d.* was expended, on the 13th of April last, in the purchase of an exchequer bill.

We, the auditors, appointed by the General Committee, have examined the accounts, and find the balance in the hands of the Treasurers to be as above stated.

(Signed) JOHN HUNTER.

R. S. WELLS.

*Sept.* 3, 1813. P. FERNANDEZ.

---

*The Form for a Petition in support of the Bill intended to be introduced in the next Session of Parliament, and which is recommended to be copied and adopted by the Country Practitioners.*

To the Honorable the Commons of the United Kingdom of Great Britain and Ireland in Parliament assembled.

The humble Petition of the undersigned Apothecaries, Surgeon-Apothecaries, and Practitioners in Midwifery, residing in ——————————*

Sheweth,

That apothecaries, surgeon-apothecaries, and practitioners in midwifery, form the great majority of the medical practitioners of England and Wales, and are very generally entrusted with the medical and surgical care of the population of the kingdom:

That none of the above branches of the medical profession can be practised with safety or benefit to the community, unless

* The blank to be filled up by inserting "the county of ———," or "city of ———," or "town of ———."

unless the practitioners acquire competent knowledge by some regular medical education:

That there is no existing law to prevent persons without any proper medical education practising in all or any of the above branches; and a great number of persons therefore, in every part of the kingdom, assume the character and exercise the functions of the apothecary, surgeon-apothecary, and practitioner in midwifery, who are wholly ignorant and utterly incompetent to the performance of the duties of the profession, whereby the safety and health of the community is endangered, the general character of the profession disgraced and brought into disrepute, and the interests of your petitioners greatly injured:

That it is essential to the preservation of the character of the profession, and to the interest of the community at large, that provision should be made for remedying the above evils:

That a Bill is pending in Parliament before your Honorable House, entitled "A Bill for regulating the Practice and Profession of Apothecaries, Surgeon-Apothecaries, and Practitioners in Midwifery, throughout England and Wales:"

That your petitioners verily believe if the said Bill should be passed into a law, that the evils and inconveniencies complained of by your petitioners will be remedied:

Your petitioners therefore humbly pray that the said Bill may be passed into a law, under such regulations and restrictions, and in such manner, as to this Honorable House may seem meet:

And your petitioners shall ever pray, &c.*

*(The Appendix to this Report in our next.)*

* No names can be affixed to the petition by proxy.

